# New insights into the interactions between *Blastocystis*, the gut microbiota, and host immunity

**DOI:** 10.1371/journal.ppat.1009253

**Published:** 2021-02-25

**Authors:** Lei Deng, Lukasz Wojciech, Nicholas R. J. Gascoigne, Guangneng Peng, Kevin S. W. Tan

**Affiliations:** 1 Laboratory of Molecular and Cellular Parasitology, Healthy Aging Programme and Department of Microbiology and Immunology, Yong Loo Lin School of Medicine, National University of Singapore, Singapore; 2 The Key Laboratory of Animal Disease and Human Health of Sichuan Province, College of Veterinary Medicine, Sichuan Agricultural University, Chengdu, Sichuan, China; 3 Immunology Programme and Department of Microbiology and Immunology, Yong Loo Lin School of Medicine, National University of Singapore, Singapore; University of Pennsylvania, UNITED STATES

## Abstract

The human gut microbiota is a diverse and complex ecosystem that is involved in beneficial physiological functions as well as disease pathogenesis. *Blastocystis* is a common protistan parasite and is increasingly recognized as an important component of the gut microbiota. The correlations between *Blastocystis* and other communities of intestinal microbiota have been investigated, and, to a lesser extent, the role of this parasite in maintaining the host immunological homeostasis. Despite recent studies suggesting that *Blastocystis* decreases the abundance of beneficial bacteria, most reports indicate that *Blastocystis* is a common component of the healthy gut microbiome. This review covers recent finding on the potential interactions between *Blastocystis* and the gut microbiota communities and its roles in regulating host immune responses.

## Introduction

*Blastocystis* belongs to the stramenopile group and is a common single-celled intestinal parasite of humans and a wide range of animals. It is estimated that this parasite has colonized 1 to 2 billion people worldwide, based on epidemiological surveys [1]. Based on analyses of the small subunit (SSU) rRNA gene of *Blastocystis*, 22 subtypes, which are possibly separate species, have been identified in humans and in a variety of animals [2]. Among them, ST1-9 and 12 have been reported in humans, but ST1-4 are the most common, accounting for more than 90% of human *Blastocystis* strains [3]. Interestingly, the prevalence of subtypes among regions seems to vary greatly (for a review, see [4]), and different subtypes exhibit remarkable differences in biology, such as drug resistance, immune response, pathogenicity, and effects on microbiota [5–7].

The development and application of next-generation sequencing (NGS) technologies have enabled a better understanding of the role of *Blastocystis* in the context of the gut microbiome. Recent microbiome studies indicated that this parasite can colonize the human gut for long periods of time without provoking symptoms [8] and that *Blastocystis* carriers have higher bacterial diversity than non-*Blastocystis* carriers [9–11], suggesting that it should be considered a commensal rather than a pathogen. In contrast, other studies suggested that *Blastocystis* is commonly associated with irritable bowel syndrome (IBS) and inflammatory bowel disease (IBD) [12,13]. However, most of these studies have not thoroughly analyzed the etiological role of *Blastocystis* in the development of IBS or IBD and, in fact, some IBS and IBD patients are not *Blastocystis* carriers, which in itself should cast doubt on the etiological role of *Blastocystis* in these syndromes.

### Interactions between *Blastocystis* and gut microbiota

The human gut microbiota consists of bacteria, fungi, archaea, and viruses, as well as single-celled eukaryotes [14]. These microbes have a profound impact on human health, metabolism, and the development of the immune system. The majority of human gut studies have focused on the more abundant bacterial components, even though homeostasis in the intestine is maintained through communication and interaction with a variety of microorganisms, such as eukaryotes [15]. Of particular interest is the relationship between single-celled eukaryotes, such as *Blastocystis*, with the gut microbiota, which has been an emerging research focus in recent years (Table 1).

**Table 1:** Studies on associations between *Blastocystis* and gut microbiota compositions.

Subtype	Method	Gut microbiota composition shift^a^	References
*Blastocystis* (ST1-4 and 6)	Metagenomics	INC bacterial richness; *Blastocystis* mainly found in individuals with *Prevotella* and *Ruminococcus* enterotypes	[16]
*Blastocystis*	Real-time PCR	INC in *Prevotella* and DEC *Bacteroides* and clostridial cluster XIVa	[17]
*Blastocystis*	Amplicon-based NGS	INC bacterial diversity; INC in Clostridia and Mollicutes (classes), DEC in Bacilli (class); INC in Clostridiales (order), DEC in Lactobacillales (order); INC in Ruminococcaceae and Prevotellaceae (families), DEC in Enterococcaceae, Streptococcaceae, Lactobacillaceae, and Enterobacteriaceae (families)	[18]
*Blastocystis*	Metagenomics	INC in *Clostridiales*, Firmicutes, and archaeal organisms (*Methanobrevibacter smithii*); DEC in *Bacteroides* and Proteobateria	[19]
*Blastocystis* (ST1-8)	Amplicon-based NGS	Not significant	[20]
*Blastocystis* (ST1-4, 8)	Metagenomics	INC *Sporolactobacillus* and *Candidatus carsonella*; DEC in *Bacteroides*	[21]
*Blastocystis* (ST2-3)	Amplicon-based NGS	INC bacterial richness; INC in *Prevotella copri*, *Ruminoccoccus bromii*, *Debaryomyces hansenii*, *Mucor mucedo*, *Aspergillus flavus*, *Mucor racemosus*, and *Issatchenkia terricola*; DEC in *Hymenolepis nana*	[22]
*Blastocystis* (ST1-4, 7, 8)	Amplicon-based NGS	INC bacterial diversity and richness; DEC prevalent in *Bacteroides* enterotyped samples; ST4 was more prevalent in Ruminococcaceae enterotyped samples and associated with *Akkermansia*; ST3 is inverse	[11]
*Blastocystis* (ST7)	Real-time PCR	DEC in *Lactobacillus* and *Bifidobacterium*	[6]
*Blastocystis* (ST1-4)	Real-time PCR	DEC in *Faecalibacterium prausnitzii* in males; DEC in *Bifidobacterium* sp. in males with IBS type C	[23]
*Blastocystis*	Real-time PCR	INC in *F*. *prausnitzii*/*Escherichia coli* ratio	[24]
*Blastocystis*	Amplicon-based NGS	INC bacterial diversity; INC in Firmicutes, Elusimicrobia, Lentisphaerae, and Euryarchaeota (phylum); INC in *F*. *prausnitzii* and *Roseburia* sp.; DEC in Actinobacteria, Proteobacteria, unassigned bacteria, and Deinococcus–Thermus	[10]
*Blastocystis*	Amplicon-based NGS	INC bacterial diversity; INC in *Clostridiales vadin* BB60; DEC in *Bacteroidaceae* and *Escherichia–Shigella*	[9]
*Blastocystis* (ST1-4, 7)	Amplicon-based NGS	INC bacterial diversity; INC in *Prevotella*, *Methanobrevibacter*, and *Ruminococcus*; DEC in *Bacteroides*	[25]

^a^ Comparison of individuals that were *Blastocystis* positive with individuals that were *Blastocystis* negative.

DEC, decrease; INC, increase; NGS, next-generation sequencing.

### The beneficial associations of *Blastocystis* on gut microbiota

A retrospective metagenomics approach to studying *Blastocystis* first revealed that the presence of *Blastocystis* was positively associated with the *Prevotella*- or *Ruminococcus*-driven enterotype and higher bacterial richness, whereas individuals with intestinal microbiota dominated by *Bacteroides* were much less likely to carry *Blastocystis* [16]. Moreover, the same research team verified these findings by analyzing another set of fecal samples, observing that individuals who were *Blastocystis*-colonized alone or along with *Dientamoeba fragilis* had relatively low abundance of *Bacteroides* and Clostridial cluster XIVa and high levels of *Prevotella* [17]. Another large-scale comparative metagenomics study of *Blastocystis* was recently conducted by Beghini and colleagues, where the presence of *Blastocystis* was negatively associated with *Bacteroides* and Proteobateria, whereas strong co-occurrence with Clostridiales, Firmicutes, and archaeal organisms (especially *Methanobrevibacter smithii*) was observed [19]. The increase of *Bacteroides* seems to be associated with lower bacterial diversity [26], colorectal cancer [27,28], celiac disease [29], and low-grade inflammation [30], showing that colonization with *Blastocystis* may be related to a healthy gut microbiota.

Interestingly, recent findings showed that colonization of *Blastocystis* was strongly associated with increased bacterial richness and various shifts in composition of the gut bacterial microbiota (Table 1). Tito and colleagues showed that the presence of *Blastocystis* was linked to microbial richness and diversity and found that *Blastocystis* was less prevalent in *Bacteroides* enterotyped samples [11]. Similarly, *Blastocystis*-colonized patients exhibited a higher bacterial diversity and a higher abundance of the *Clostridia* class, Ruminococcaceae, and Prevotellaceae families, as well as *Faecalibacterium* and *Roseburia* genera, while Enterobacteriaceae were enriched in *Blastocystis*-free patients [18]. Enterobacteriaceae, a family of large gram-negative bacteria, are typically found in a higher abundance in IBD [31]. A similar observation was also recorded by Kodio and colleagues who also observed increase in *Faecalibacterium prausnitzii* and *Roseburia* sp. in *Blastocystis*-colonized children [10]. The *Faecalibacterium* and *Roseburia* genera are able to produce butyrate, which is one of the most important metabolites for maintaining colonic health and is the major energy source of colonic epithelial cells [32]. Additionally, butyrate can induce the differentiation of T regulatory (Treg) cells via up-regulation of the Foxp3 gene, to suppress inflammatory and allergic responses [33].

Another study compared the relationship between 3 common intestinal parasites (*Blastocystis*, *Giardia duodenalis*, and *Entamoeba* spp.) and gut microbiota composition in humans. Interestingly, *G*. *duodenalis*–positive samples were correlated with a low *F*. *prausnitzii*/*Escherichia coli* ratio, while *Blastocystis* and *Entamoeba* spp.–positive individuals related to a higher *F*. *prausnitzii*/*E*. *coli* ratio [24]. *F*. *prausnitzii* is able to reduce the production of the pro-inflammatory cytokines interleukin (IL)-12 and tumor necrosis factor alpha (TNFα) in vitro studies using peripheral blood lymphocytes-derived dendritic cells (DCs) [34]. In addition, *Blastocystis* was associated with high bacterial diversity and with *Clostridiales vadin* BB60, while the individuals without *Blastocystis* had a higher abundance of Bacteroidaceae and *Escherichia*–*Shigella* [9]. A previous study reported that high abundance of *Escherichia*–*Shigella* appeared to reduce the bacterial diversity and was associated with pro-inflammatory effects [35]. However, other studies revealed no significant bacterial composition differences between *Blastocystis*-positive and *Blastocystis*-negative IBS patients [20].

Relationships between *Blastocystis* and eukaryotic microbiota have also been demonstrated in recent years. Nieves-Ramírez and colleagues [22] evaluated the fecal bacterial and eukaryotic microbiota from 156 asymptomatic adult subjects by amplification and sequencing of the 16S rRNA gene and 18S rRNA genes, respectively, and further compared the composition differences between *Blastocystis*-colonized and *Blastocystis*-free individuals. *Blastocystis* carriers had a higher abundance of *Prevotella copri* and *Ruminococcus bromii*, which are commonly found in the human gut microbiome and are often described as either *Prevotella*-rich or *Ruminococcus*-rich [36]. *Blastocystis* colonization was also associated with increases in yeast and fungal species (*Debaryomyces hansenii*, *Mucor mucedo*, *Aspergillus flavus*, *Mucor racemosus*, and *Issatchenkia terricola*) and a decrease of *Hymenolepis nana* [22]. *H*. *nana* is a one of the most common cestodes in the human gut and is often associated with pathological consequences [37].

In general, these studies revealed that the presence of *Blastocystis* was associated with higher gut bacterial diversity and negatively correlated with the levels of *Bacteroides*. As the higher bacterial diversity is commonly associated with health and lower incidence of inflammatory diseases [38] and the *Bacteroides* community type has been linked to obesity, inflammation of the lower gastrointestinal tract, and reduced microbiota resilience [39], it suggests that *Blastocystis* colonization is associated with a healthy gut microbiome.

### The adverse associations of *Blastocystis* on gut microbiota

Apart from these studies that propose a commensal role for *Blastocystis*, the pathogenic potential of *Blastocystis* has also been reported (for a review, see [40]). Nourrisson and colleagues suggested that the level of *Bifidobacterium* sp. was decreased in *Blastocystis*-colonized male IBS type C patients (IBS patients with constipation based on the Rome III classification [41]) and that healthy *Blastocystis*-positive individuals had a significant decrease in *F*. *prausnitzii* [23]. Similarly, a more recent mouse model study in our laboratory revealed that *Blastocystis* can decrease the abundance of beneficial bacteria *Bifidobacterium* and *Lactobacillus* [6]. *Bifidobacterium* are known as protective bacteria that have anti-inflammatory properties [42]. Although some strains from *Lactobacillus* have been linked to sepsis, especially in immunocompromised hosts [43], bacteria belonging to *Lactobacillus* genus have also been used to prevent opportunistic pathogens infection in the gastrointestinal tract [44]. *Blastocystis* ST4 colonization in rats can increase the relative abundance of *Oscillospira* and decrease the level of *Clostridium*, which is linked to lower amounts of short-chain fatty acids (SCFAs) [45]. In addition, Vega and colleagues showed a significant association between the presence of *Blastocystis* and *Clostridium difficile* infection in diarrhea patients [46]. Overall, although these studies reported that the presence of *Blastocystis* can reduce the abundance of beneficial bacteria, leading to a dysbiotic state, the etiological role of *Blastocystis* in the development of gastrointestinal diseases, especially IBS, needs further investigation.

### Associations between specific *Blastocystis* subtypes and gut microbiota

There are currently 22 subtypes of *Blastocystis*, which are widely distributed across human and animals, with some subtypes specific to 1 group but not the other [3] (Table 2). Notably, in linking specific subtypes of *Blastocystis* to intestinal microbes, only a few subtypes have been well-documented when studying the interactions with gut microbes. For instance, the presence of ST4 in Swedish travelers was associated with higher abundances of the bacterial genera *Sporolactobacillus* and *Candidatus carsonella*, while ST3 did not show such significant relationships [21]. Similarly, ST3 and ST4 showed inverse (negative and positive, respectively) correlations to *Akkermansia* abundance in fecal samples from Flemish gut Flora Project (FgFP) [11], a bacteria that has been linked to delayed onset of obesity and its associated metabolic disorders in murine models [47]. A more recent study showed that ST3 was accompanied by potentially beneficial species, such as *Prevotella*, *Methanobrevibacter*, and *Ruminococcus* [25], whereas co-incubation with lactic acid bacteria has been shown to exhibit strong inhibitory effects on *Blastocystis* ST3 proliferation in vitro [48]. However, Yason and colleagues showed that presence of ST7 is associated with a decrease of the beneficial bacteria *Lactobacillus* and *Bifidobacterium* in a mouse model [6]. Collectively, these findings suggest differential associations between subtypes and host gut microbiota. In light of the enormous inter-genetic variation between different subtypes that exhibited different or even opposite effects [49,50] and only limited studies investigating links between *Blastocystis* subtypes to gut microbes, future studies should focus on the potential link among *Blastocystis* and both microbiota community structure and host health, at subtype resolution.

**Table 2:** The known hosts for various subtypes of *Blastocystis*.

Subtypes	Hosts	References^a^
ST1	**Human**; Nonhuman primates; Cattle; Pig; Goat; Sheep; Dog; Cat; Rodents; Birds; Raccoon; Fish; Captive wild animals	[3,51]
ST2	**Human**; Nonhuman primates; Pig; Dog; Rodents; Birds; Raccoon; Fish; Captive wild animals	[3,51,52]
ST3	**Human**; Nonhuman primates; Cattle; Pig; Goat; Sheep; Dog; Cat; Rodents; Horse; Raccoon; Captive wild animals	[3,51]
ST4	**Human**; Nonhuman primates; Cattle; Goat; Dog; Cat; Rodents; Rabbit; Birds; Marsupials; Captive wild animals	[3,51]
ST5	Human; Nonhuman primates; **Pig**; Cattle; Goat; Sheep; Dog; Rodents; Birds; Captive wild animals	[53]
ST6	Human; Cattle; Pig; Goat; **Birds**	[54]
ST7	Human; Nonhuman primates; Goat; Dog; Rodents; **Birds**; Fish	[52,54]
ST8	Human; **Nonhuman primates**; Marsupials; **Dog**; Birds; Fish	[51,52]
ST9	Human	[54]
ST10	Nonhuman primates; **Cattle**; **Sheep**; Pig; Goat; Dog; Cat; Birds; Marsupials; Fish; Captive wild animals	[52]
ST11	Nonhuman primates; Elephant	[55]
ST12	Human; Cattle; Marsupials; Captive wild animals	[51]
ST13	Nonhuman primates; Marsupials; Captive wild animals	[55–57]
ST14	Cattle; Goat; Sheep; Cat; Captive wild animals	[56,57]
ST15	Nonhuman primates; Sheep; Water Vole	[57]
ST16	Marsupials	[58]
ST17	Cattle; Rodents	[51]
ST21	Waterbuck; Cattle	[56,59]
ST23	Cattle	[59,60]
ST24	Cattle; Sheep; Lama glama	[2,59,60]
ST25	Cattle; Sheep	[2,59,60]
ST26	Cattle; Sheep	[2,59,60]

^a^ Some references are from reviews because the same subtype has been identified in a wide range of hosts in many studies.

Bold represents the dominant host.

### Interactions between *Blastocystis* and the immune system

The mechanisms of interaction between eukaryotic parasites and the host have long been the focus of research [61]. Coevolution of gut parasites with the mammalian immune system has promoted the development of complex parasite–host interactomes. In short, these interspecies ties may reveal mutualistic, commensal, or parasitic character alongside many intermediate scenarios, with no explicit cutoff defining the outcome of the host–parasite synergy. Although *Blastocystis* constitute the most common human-related protist, potentially beneficial or detrimental impacts of these parasites on the host immune system are still under debate [62].

Human-associated *Blastocystis* represent a genetically diverse component of the gut microbiome [63]. Our previous research demonstrated intra- and inter-subtype variability in terms of ST4 and ST7 pathogenicity [50]. The genetic diversity within the *Blastocystis* genus is plausibly a critical factor that dictates colonization susceptibility and determines interaction outcomes with the gut immune system. New insights into the *Blastocystis*–host interactome came from sequencing of the *Blastocystis* genome. The comparison of ST1-, ST4-, and ST7-derived genomes revealed considerable variation in respect to the genomes’ assembly size, guanine-cytosine (GC) pair content, and the number of protein-coding genes [49]. Proteases constitute an important component of the *Blastocystis* secretome. Beside engagement in many essential biological processes, proteases are suspected to be potential virulence factors [64]. Importantly, the number and type of protease genes among the subtypes varies greatly [49]. This could provide a possible explanation for the variable clinical significance of *Blastocystis*.

Most immunological studies on the interactions between *Blastocystis* and host immune system have focused on ST4 and ST7. The zoonotic subtypes ST4 and ST7 isolates were originally isolated from a Wistar rat and a patient with gastrointestinal symptoms in Singapore, respectively [65,66]. ST4 is the most common subtype in European individuals based on metagenomic studies of the human gut [11,19], while ST7 is rarely found in populations although it was isolated from human. Therefore, we need to note that studies of ST7’s potential pathogenicity may tell us little about how the majority of *Blastocystis* subtypes present in human gut, for example, ST1 and ST3, and to a lesser extent ST2 and ST4, interact with the human immune system.

**Fig 1:**
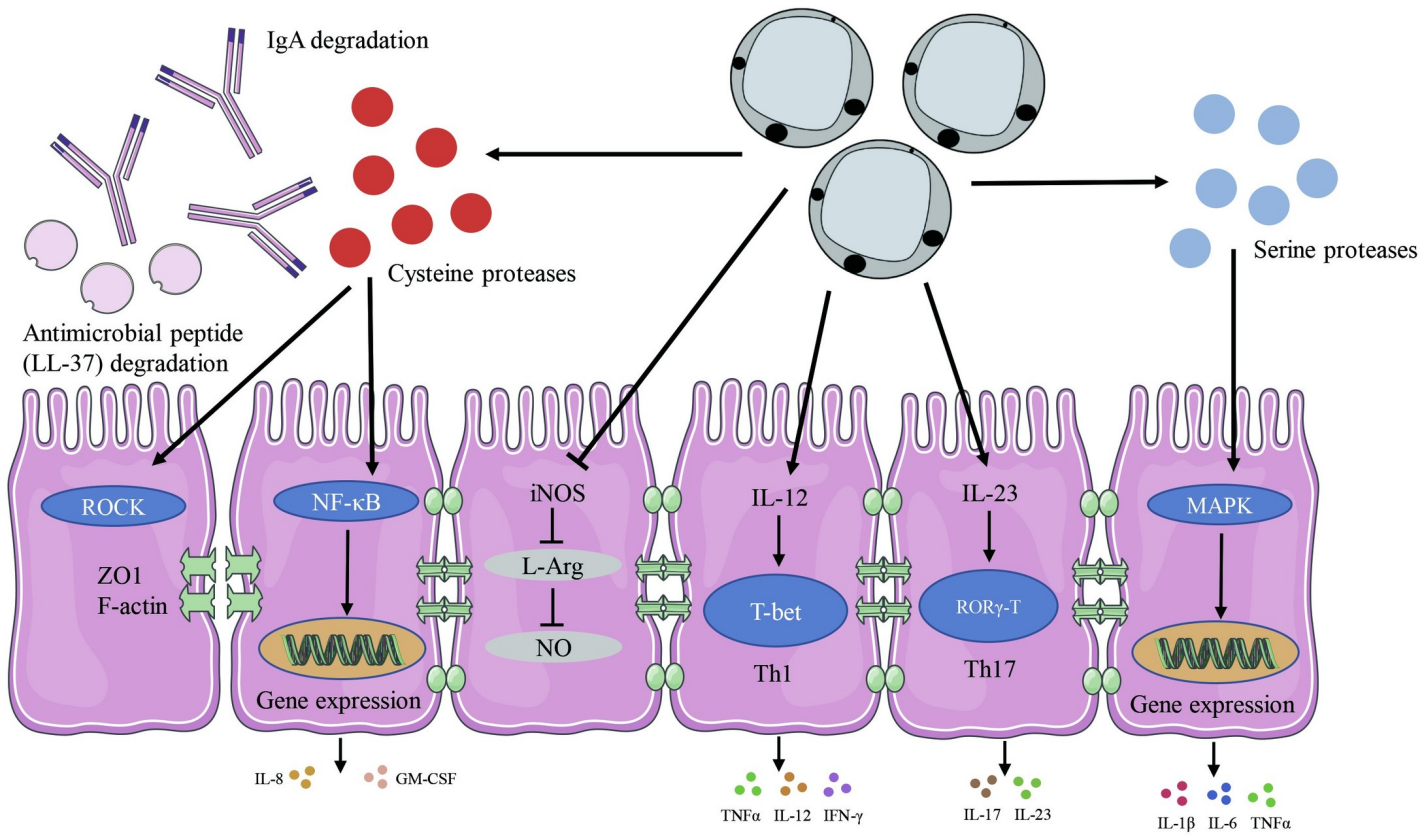
*Blastocystis*-mediated regulation of immune responses and homeostasis as characterized by studies using in vitro systems and experimental rodent models. This illustration simplifies the many interactions and pathways involved in *Blastocystis* colonization or infection in host cells. Cysteine proteases produced by *Blastocystis* are able to degrade IgA and AMP (LL-37). *Blastocystis* can also influence the gene expression of pro-inflammatory cytokines by regulating the NF-κB and MAPK pathways. *Blastocystis* evades host NO antiparasitic response by inhibiting iNOS to convert arginine to NO. *Blastocystis* can induce Th1 and Th17 cells responses and their signature cytokines release. AMP, antimicrobial peptide; GM-CSF, granulocyte-macrophage colony-stimulating factor; IFNγ, interferon gamma; IgA, immunoglobulin A; IL, interleukin; iNOS, inducible nitric oxide synthase; L-Arg, L-Arginine; MAPK, mitogen-activated protein kinase; NF-κB, nuclear factor-κB; NO, nitric oxide; Th, T helper; TNFα, tumor necrosis factor alpha; ZO1, zonula occludens-1.

Some in vitro studies over the past few decades have been designed to investigate the effects of *Blastocystis* on host intestinal cells (Fig 1). Cathepsin B, a cysteine protease produced by ST7, has been linked to increased Caco-2 cell monolayer permeability [67]. The trans-epithelial permeability was regulated by tight junctions, which play an essential role in controlling the polarization of epithelial cells and protecting deeper tissues from external microbial pathogen infections [68]. Our previous in vitro study indicated cysteine proteases produced by ST7 induce zonula occludens-1 (ZO-1) and F-actin compromise, in a rho-kinase (ROCK)-dependent manner, in intestinal epithelium [69]. Similarly, ST4 also has the ability to increase the epithelial permeability in IEC-6 cell monolayers [70]. The intestinal permeability of patients with *Blastocystis* infection was also significantly higher than that of healthy individuals [71]. It is worth noting that the effects of *Blastocystis* on intestinal cells are mainly based on in vitro studies and may have little bearing on what happens in the human intestine.

### Interactions between *Blastocystis* and the innate immune system

The innate immune system constitutes the first line of host defense and plays a crucial role in preventing microbial pathogen infection, while tolerating the normal host flora [72]. Long and colleagues reported that incubation with ST1 modulated the immune response by stimulating the release of the cytokine IL-8 in vitro [73]. IL-8 is a chemokine regulating neutrophil movement as well as, to a lesser extent, other granulocytes [74]. More detailed, mechanistic studies revealed that cysteine proteases produced by ST4-WR1 activate IL-8 gene expression in human colonic epithelial T84 cells in a nuclear factor-κB (NF-κB)-dependent process [75]. Another feature of *Blastocystis* ST7-B and ST4-WR1-derived cysteine proteases is an ability to degrade immunoglobulin A (IgA), a major immunoglobulin class involved in mucosal defense [76]. Thus, the secretion of cysteine proteases might constitute a parasite’s adaptation mechanism, facilitating colonization by protecting protists in the intestinal milieu against the host’s immune system.

One of the most ancient elements of innate immunity is represented by antimicrobial peptides (AMPs). LL-37 is a 37-amino acid fragment of the human AMP cathelicidin, which exhibits antimicrobial and immunomodulatory activity [77]. In a recent study, employing mouse intestinal explants and the human intestinal epithelial cell line HT-29, it was shown that *Blastocystis* (ST1, ST4, and ST7) are able to induce intestinal epithelial cells to secrete LL-37 [78]. ST1 and ST4 are susceptible to the effects of LL-37, while ST7 was resistant to the cytotoxic effects of LL-37 through secretion of proteases to degrade LL-37 and an acidified environment to attenuate LL-37 activity [78].

Toll-like receptors (TLRs) are a major family of pattern recognition receptors (PRRs) that play an essential role for protective immunity against infection. TLRs activate downstream signaling cascades which mediate the activation of transcription factors such as NF-κB [79]. In a recent in vitro study, *Blastocystis* exhibited pleiotropic effects in the modulation of TLR activation by specific ligands (zymosan, lipopolysaccharide (LPS), and flagellin) [80]. Specifically, *Blastocystis* ST7-B and ST4-WR1 significantly inhibited zymosan-mediated NF-κB activation in human TLR reporter monocytic cell line (THP1-Blue), and neither subtype had any significant effect on flagellin-mediated activation [80]. Interestingly, *Blastocystis* ST4-WR1 was able to inhibit LPS-mediated NF-κB activation in THP1-Blue cells, while ST7-B was found to augment the effect of LPS-mediated NF-κB activation [80]. However, it should be noted that these results only observed in vitro systems, and future studies should focus on human hosts or other naturally colonized hosts to better understand the role of *Blastocystis* in host innate immunity.

*Blastocystis* ST7, but not ST4, induces strong expression of pro-inflammatory cytokines IL-6, IL-1β, and TNFα in murine macrophages mediated by mitogen-activated protein kinases (MAPKs) [7]. The MAPK cascade is one of the most important and evolutionarily conserved signaling pathways and plays an essential role in innate immunity [81]. The MAPKs of mammals can be divided into 3 main families: the extracellular signal-regulated kinases (ERKs), c-jun NH_2_-terminal kinases (JNKs), and p38 MAP kinases [81]. *Blastocystis* ST7 triggered ERK and JNK pathways to regulate the expression of pro-inflammatory cytokines in macrophages in vitro, including IL-1β, IL-6, and TNFα [7]. Interestingly, *Blastocystis*-derived serine proteases increase the pro-inflammatory cytokine expression in a MAPK-dependent manner, while cysteine proteases were able to induce these pro-inflammatory cytokines in a MAPK-independent pathway [7].

Nitric oxide (NO), an endogenously produced molecule, constitutes an important element of the innate intestinal response against luminal pathogens [82]. NO has a strong antimicrobial activity by reacting with a large spectrum of molecules such as DNA, proteins, and lipids [82]. In vitro experiments revealed that ST7-B but not ST4-WR1 is able to inhibit NO production, and this was associated with down-regulation of the transcriptional expression of host cell inducible nitric oxide synthase (iNOS) [83]. Although *Blastocystis* inhibits an intestinal epithelial NO response in vitro, whether the effect happens in the intact human intestine or in a human intestine damaged by other pathogens (bacterial or viral or parasitic) needs further investigation.

### Interactions between *Blastocystis* and the adaptive immune system

In addition to the impact on innate immune function, recent research also uncovered that *Blastocystis* plays a role in the adaptive immune system. T helper 1 (Th1) and T helper 2 (Th2) cells and their signature cytokines (e.g., interferon gamma (IFNγ), TNFα, and IL-12 for Th1 and IL-4, IL-5, and IL-13 for Th2 lineage) are important for protection against parasites, intracellular and extracellular pathogens, such as viruses and bacteria [84]. At the same time, potent Th1 or Th2 response at the site of infection may overwhelm the regulatory compartment, which in consequence might lead to chronic inflammation [85]. Yoshikawa and colleagues demonstrated that upon *Blastocystis* ST4 colonization, rats did not show any pathological lesions; however RNA transcripts for IFNγ, IL-12, and TNFα underwent substantial increases in intestinal tissue of colonized rats [86]. Interestingly, *Blastocystis* ST4 chronic colonization induces noninflammatory colonic hypersensitivity in rats [45]. Importantly, the colonic levels of IL-6, lipocalin-2 proteins, and GATA-3, a Th2 lineage master regulator, were not changed significantly in *Blastocystis*-colonized rats [45]. Thus, all these reports indicate a rather controlled engagement of these 2 T helper lineages in an immune response in the context of *Blastocystis* ST4.

Another more recently identified component of the intestinal T helper compartment comprises CD4 T cells expressing RORγ-T and producing the cytokine IL-17. RORγ-T is a key transcription factor for promoting T cells differentiation into T helper 17 (Th17) cells [87]. IL-17 is known as the signature cytokine of Th17 cells, a CD4 subset contributing to the pathogenesis of various inflammatory diseases [88]. What is important is that the recruitment of CD4 cells toward conventional Th17 phenotype occurs in peripheral organs particularly in the large intestine [89,90]. Furthermore, the site of Th17 lineage polarization does not define a terminal niche for this subset as these cells after differentiation can migrate to the distal locations in respect to the organs of Th17 origin [91,92]. Hashimoto’s thyroiditis (HT) represents an autoimmune disorder with a Th17 lineage playing an essential role in thyroid’s pathogenesis [93,94]. El-Zawawy and colleagues investigated the relationship of *Blastocystis* and IL-17 in patients with HT. *Blastocystis*-colonized HT patients had more IL-17 compared to *Blastocystis*-free HT patients, whereas the amount of IL-17 was significantly decreased after *Blastocystis* eradication [95]. All these observations correlate with other work in which authors showed increased IL-17 and IL-23 expression in the intestinal mucosa of mice colonized with *Blastocystis* [96]. Although these 2 studies lack information regarding *Blastocystis* subtypes, the patterns of cytokine makeup in *Blastocystis*-colonized HT patients and in intestines of *Blastocystis*-colonized rodents indicate the potential links between some subtypes and generation of pro-inflammatory Th17 compartment. Hence, functional remodeling of gut microbiomes upon particular *Blastocystis* subtype colonization or direct interaction of these protists with a host CD4 T immune compartment might lead to the enhanced development and induction of the Th17 pro-inflammatory subset.

In summary, *Blastocystis* appears to interact with the host immune system at several levels (Fig 1). Although some reports demonstrate that *Blastocystis* colonization might result in Th1 and Th17 cell responses, the precise mechanism and role of these lineages in controlling colonization-associated pathogenicity has not been clearly explained. The microbiome plays a pivotal role in the shaping and development of the host’s innate and adaptive immune system [97]. Alterations in the composition of the commensal microbiota can influence the frequency of mucosal Treg cells [98]. Indeed, the genus *Clostridium*, particularly clusters IV and XIVa, can promote accumulation of Tregs, and *Clostridium*-colonized mice markedly enhanced the differentiation of Foxp3-expressing cells [99]. The immune changes induced by *Blastocystis* colonization may directly influence the host gut microbiota composition. On the other hand, the remodeling of the intestinal microbiome upon *Blastocystis* colonization may indirectly modulate the host immune system. Therefore, the complex interactions of the *Blastocystis* microbiome-immune network requires in-depth studies, which are currently lacking, for mechanistic insights into their roles in gut health and disease.

## Conclusion and future perspectives

Based on the above summary of work carried out on *Blastocystis*, the evidence overwhelmingly points to *Blastocystis* as a commensal, although a rare subtype (ST7) has shown pathogenicity in in vitro systems and experimental rodent models. Several in vitro and a handful of in vivo rodent experiments reveal that that the common ST4 exhibits mild inflammation and pathology to host tissues. However, we cannot simply regard or designate *Blastocystis* as a commensal considering numerous genetic variations among subtypes. More microbiome and immunological research should be conducted on humans or other natural hosts at the subtype level to determine if it is a commensal or a pathogen. Furthermore, the mechanisms of the interactions between *Blastocystis* and gut microbiota are still relatively poorly defined. Since it has been determined that *Cryptosporidium* infections can affect the metabolite profiling in mouse model [100,101], it would appear appropriate to also ascertain whether *Blastocystis* colonization is able to affect gut microbial-derived metabolites, such as SCFAs, bile acids (BAs), tryptophan, and/or other metabolites, to maintain the host health and immune homeostasis should be the focus of future research. Importantly, *Blastocystis* research has mainly used in vitro systems and the experimental rodent models that harbor a divergent microbiota from humans. Therefore, exploring the roles of *Blastocystis* in impacting immunity requires more mechanistic studies based on human hosts or in other animals with natural colonization.
